# Processing of End-of-Life Materials and Industrial Wastes

**DOI:** 10.3390/ma15217662

**Published:** 2022-10-31

**Authors:** Ndue Kanari, Seit Shallari, Eric Allain

**Affiliations:** 1 Université de Lorraine, CNRS, GeoRessources, F-54000 Nancy, France; 2 Faculty of Agriculture and Environment, Agricultural University of Tirana, 1029 Tirana, Albania

**Keywords:** industrial wastes and by-products, end-of-life materials, EAF and blast furnace slags, WEEE and PCBs, mining waste, bauxite residue, waste glass, livestock waste, valuable, critical, and strategic elements, thermochemical and combined processing methods

## Abstract

This Special Issue (SI) offered the opportunity to present the latest scientific developments and findings in the field of processing of end-of-life materials and solid industrial wastes. Due to the large quantity of wastes generated and to their complex elemental and mineralogical composition, the approaches, methods and processes proposed for their decontamination, energy beneficiation and high-added-value metal recovery are complex and diverse. Some transversal research investigations using wastes as remediation agents and for synthesis of new materials were also included in the SI. After a brief introduction, the main scientific contributions and findings of each article published in the SI are summarized.

## 1. Introduction

Despite scientific and technical advances in extractive metallurgy, materials manufacturing and other industrial sectors, the generation of by-products and wastes is inevitable. In addition, along with the depletion of raw materials rich in valuable substances, the quantity of solid residues generated will increase in the future. However, their content in elements considered critical and strategic [1,2,3] often greatly exceeds that found in natural deposits. Other by-products are almost pure and they are used for the synthesis of new materials [4,5] utilized for water and wastewater treatment. Another source of metals of interest are end-of-life materials, such as electrical waste and electronic equipment, known as WEEE, representing a vast and constantly increasing waste stream [3]. In the dynamics of a circular economy, sustainable development, environmental and societal imperatives, as well as the shortage of natural deposits rich in metals, waste material is progressively becoming an attractive target for research orientation. The challenge remains to understand and develop concepts, methods and techniques to design, optimize and manage processes that extract and recover the useful part of end-of-life materials and wastes.

In this context, this Special Issue (SI) is an international forum (authors and/or co-authors from Albania, Australia, Chile, China, France, Mexico, Poland, Romania and Russia) exposing scientific developments in the field of processing of end-of-life materials and solid industrial wastes. The scientific and innovative approaches and advanced understanding in different methods for the processing of complex residual materials are developed and documented in 17 research papers of this SI, named “Processing of End-of-Life Materials and Industrial Wastes” (https://www.mdpi.com/journal/materials/special_issues/processing_materials_wastes (accessed on 20 October 2022)).

Main scientific contributions and findings, shortly described for each article published in this SI, are available in the next Section.

## 2. Short Description of the Articles Published in This Special Issue

Menad et al. [6] authored an article, entitled “New EAF Slag Characterization Methodology for Strategic Metal Recovery”. Generated from electric arc furnace (EAF), this material represents a significant potential economic resource due to its high content in strategic metals, such as molybdenum, vanadium and chromium. A well-established protocol was applied for the physico-chemical characterization of the steel slag prior selecting the processing method consisting of the roasting process of the co-grinding the EAF slag with alkali metal reagents (NaOH, KOH) at temperatures approaching 800 °C. Over 96% of chromium and molybdenum are transformed, respectively, into chromate (CrO_4_^2−^) and molybdate (MoO_4_^2−^) of alkali metals that are separated from the roasted slag by leaching in water.

Deng et al. [7] contributed with an article, entitled “Effect of Waste Glass on the Properties and Microstructure of Magnesium Potassium Phosphate Cement”. Huge amounts of waste glass, including, especially, flat glass, daily glassware and glass packaging containers, are discarded in the world. The effect of glass powder (GP) on the mechanical and working properties of magnesium potassium phosphate cement (MKPC) is evaluated. The addition of glass powders (at less than 40%) improved the mechanical properties of MKPC and reduced the heat of hydration. The alkali components in the GP participated in the reaction of MKPC and the hydration products were closely bound to the matrix of the magnesium phosphate cement.

A research work, entitled “Improving the Properties of Degraded Soils from Industrial Areas by Using Livestock Waste with Calcium Peroxide as a Green Oxidizer”, is reported by Więckol-Ryk et al. [8]. The treatment and use of livestock waste has posed a significant problem in environmental engineering. A new approach to application of calcium peroxide (CaO_2_) as a green oxidizer and microbiocidal agent in the treatment of poultry manure is developed. The antimicrobial effect is linked with releasing active oxygen without producing any harmful substances, which makes CaO_2_ an ecologically friendly compound. Adding CaO_2_ as an amendment to poultry manure has a positive effect on germination and growth of grass seed mixtures, improves the quality of the soils and groundwater and may be used for soil reclamation in industrially degraded areas.

A comprehensive study by Kanari et al. [9], entitled “Some Aspects of the Thermochemical Route for the Valorization of Plastic Wastes, Part I: Reduction of Iron Oxides by Polyvinyl Chloride (PVC)”, is also published in this Special Issue. Used PVC is one of the most problematic occurrences of plastic waste with a raw chemical formula (C_2_H_3_Cl)_n_ containing a high amount of chlorine (ca. 57 wt% Cl in pure PVC). The use of PVC as an alternative to classical reducing agents for the direct reduction of iron oxides is developed. The proposed original research design is composed of two steps: (i) The first step involves the study of the temperature effect on the de-chlorination extent of the PVC under air atmosphere, (ii) while the second step involves the investigation of the reducing capability of the de-chlorinated PVC for the reduction of hematite (Fe_2_O_3_) at diverse temperatures and reaction times. Temperatures equal to or less than 1100 °C were preferred, which is substantially lower than those observed during the manufacturing of cast iron and steel.

Dziki et al. [10] submitted a research work, entitled “Micronized Oat Husk: Particle Size Distribution, Phenolic Acid Profile and Antioxidant Properties”. Oat is becoming more popular as a raw material in various sectors of the food industry because of its well-known health benefits. Oat grain is rich in many bioactive compounds, such as phenolic acids, flavonoids, avenanthramides and fiber, while oat husk (OH; hull) is a by-product obtained from oat processing by the food industry. The effect of the working parameters of an impact classifier mill on the particle size distribution, phenolic profile and antioxidant properties of pulverized OH was investigated. This study demonstrated that the sterilization of OH decreases the moisture content, thereby resulting in a very dry material with appropriate microbiological purity. The proposed method of micronization effectively reduced the particle size of the sterilized husk.

Cobîrzan et al. [11] describes novel research in “Volcanic Tuff as Secondary Raw Material in the Production of Clay Bricks”. Mixtures of clay and tuff with various proportions were fired at 900 to 1100 °C. The compressive strength of tuff–clay samples depended strongly on the sintering temperature. When heated to 900 °C, the values of compressive strength were lower than 15.7 MPa, while samples fired at 1100 °C presented higher compressive strengths, an increase of up to three-fold as compared with the reference bricks. This increase was due to the creation of stronger bonds between the particles. The increase in compression strength was also due to the formation of an important volume fraction of liquid that further accelerates the sintering process. This work showed that tuff can be successfully used as a secondary raw material in the fabrication of fired clay bricks, in ratios of up to 30%.

Więckol-Ryk et al. [12] contributed a second study, entitled “Solid Peroxy Compounds as Additives to Organic Waste for Reclamation of Post-Industrial Contaminated Soils”. The solid inorganic peroxyde compounds, such as calcium peroxide (CaO_2_) and sodium percarbonate (2Na_2_CO_3_·3H_2_O_2_), are widely used as stimulators of remediation processes in soils contaminated with heavy metals and hardly biodegradable organic compounds. The use of both compounds as biocidal agents to reduce pathogens in organic fertilizers is associated with the simultaneous stimulation of root and shoot growth of test plants. The analysis of water leachates of soils treated with inorganic peroxyde compounds showed that the agents increase the bioavailability of components necessary for proper seed germination and plant growth (N, P, K, Ca, Mg and S).

Qin et al. [13] submitted their work, entitled “Effect of Oily Sludge Treatment with Molten Blast Furnace Slag on the Mineral Phase Reconstruction of Water-Quenched Slag Properties”. Blast furnace slag (BFS) is the most important by-product in iron and steel production, while the oily sludge (OS) is one of the major oily wastes that is associated with petroleum exploitation, transportation and processing, which is also a highly polluting agent and quite hard to process. The pyrolysis process of the oily sludge with the molten BFS not only effectively treats the sludge and reuses the heat of the BFS, but also solidifies the heavy metal of OS with BFS. The solidification rate decreases with the increase in oily sludge and the appropriate absorption proportion of the OS represents no more than 30%. The obtained solid product after pyrolysis can be used for the production of cement and other building materials.

Cerecedo-Sáenz et al. [14] provided a research study, entitled “Use of the O_2_-Thiosemicarbazide System, for the Leaching of: Gold and Copper from WEEE & Silver Contained in Mining Wastes”. A kinetic study of the leaching of Au and Cu contained in waste electrical and electronic equipment (WEEE) and Ag contained in mining wastes (MW), using the O_2_-thiosemicarbazide system, was performed. The activation energies (Ea) found that the leaching of WEEE is controlled by diffusion (Cu; Ea = 9.06 and Au; Ea = 18.25 kJ/mol), while the leaching of Ag (Ea = 45.55 kJ/mol) from MW is controlled by the chemical reaction. The pH has an effect only at values above 8 and for the case of MW, the effect of O_2_ partial pressure has a distinct effect, increasing the Ag leaching from 33% at 0.2 atm up to 60% at 1 atm. Results led to the possibility of using the O_2_-thiosemicarbazide system directly in leaching processes to dissolve gold, silver and copper from wastes (electronic and/or industrial).

Fosu et al. [15] described their research work, entitled “Physico-Chemical Characteristics of Spodumene Concentrate and Its Thermal Transformations”. Lithium (produced from salar brines or ores) is undergoing important investigations in order to meet a worldwide stable supply as this element is now classified as a critical metal by many countries. This study aimed to characterize a lithium concentrate (spodumene-type pegmatite deposit from Western Australia) as well as understand its thermal transformation behaviors. The thermal behavior of spodumene and the concentration of its polymorphs were studied by thermal treatments in a range of 900 to 1050 °C. All three polymorphs of the mineral (α, γ and β spodumene) were identified. Full transformation of the α-phase was achieved at 975 °C and 1000 °C after 240 and 60 min treatments, respectively. Applying first-order kinetic models to the two processes provides a satisfactory fit to the experimental data and yields kinetic parameters and apparent activation energies of 655 and 731 kJmol^−1^, respectively, for α- and γ-decay.

Qin et al. [16] submitted a second publication, entitled “Metallurgical Coke Combustion with Different Reactivity under Nonisothermal Conditions: A Kinetic Study”. The combustion characteristics and kinetics of high- and low-reactivity metallurgical cokes in an air atmosphere were studied using thermogravimetric technique and the apparent activation energy was calculated using different kinetics methods. It was established that with an increase in the heating rate, the ignition temperature and burnout temperature of the two cokes increased, the combustion time was shortened, the comprehensive combustion characteristic index increased and the combustion characteristics were improved. Low-reactivity coke had better thermal stability and combustion characteristics. The coke combustion kinetic parameters provided the basic data parameters for the numerical simulation of blast furnace pre-tuyere combustion and provided the basis for the application of high-reactivity coke in blast furnaces.

Shoppert et al. [17] contributed an article, entitled “High-Selective Extraction of Scandium (Sc) from Bauxite Residue (Red Mud) by Acid Leaching with MgSO_4_”. Bauxite residue, also known as red mud (RM), from alumina production is the most promising technogenic material for the production of scandium (Sc) and other rare-earth elements (REEs). The study examined the possibility of selective REE leaching from RM obtained by the water–alkali leaching of the electrostatic precipitator dust using MgSO_4_ as a leaching reagent. High REE extraction efficiency was obtained even under mild leaching conditions. More than 80% of Sc was extracted at pH = 2, T = 80 °C, L/S ratio of 10, C_MgSO4_ of 24 g L^−1^ and leaching time of 60 min. The Fe extraction at these conditions was 7.7%. Increasing the pH to 4 led to a decrease in Sc and Fe extraction to 63.5% and 0.03%, respectively. The dissolution of other major components was also significantly reduced at a pH of 4. Increasing the pH to 6 led to a very low extraction of REEs (<15%).

Salinas-Rodríguez et al. [18] authored a submission, entitled “Leaching of Copper Contained in Waste Printed Circuit Boards, Using the Thiosulfate—Oxygen System: A Kinetic Approach”. This research work is devoted to the treatment of crushed printed circuit board (PCB) wastes from electrical and electronic devices (WEEE), carrying out the recovery of copper in solution. Performed work is of importance due, principally, to the use of a non-toxic reagent, which will be used directly without the addition of an oxidant, such as HNO_3_, H_2_O_2_, O_3_, Cu^2+^ or Fe^2+^. In this process, the quick dissolution of Cu to Cu^2+^ and its complexation with thiosulfate, allows one to avoid the thiosulfate oxidation and, therefore, improving its recovery. The leaching reaction according to the determined kinetic values corresponded to a mixed controlled process, with, for both chemical and diffusion controls, the reaction, depending on the reagent concentration and temperature.

Tan et al. [19] contributed their research, entitled “Research on Flocculant Selection for Classified Fine Tailings Based on Micro-Characterization of Floc Structure Characteristics”. The rapid settlement of tailings is an important technical guarantee for the continuous production of downhole filling. This work research proposes to obtain samples of an optimized floc solution for classified fine tailings under single-consumption flocculant by means of an indoor static settlement experiment, including nuclear magnetic resonance observation and microscope scanning. The study also analyzes the porosity and aperture distribution characteristics of flocculation solution for tailings. The structural characteristics of floc produced in using different flocculants are characterized at a microscopic level by analyzing the flocculation size, spatial distribution characteristics, particle size characteristics and morphological characteristics of scanning electron microscope images of floc. The analysis provides a scientific basis for the selection of optimal flocculation agent from the microscopic angle.

Bazan-Wozniak et al. [20] provided research in “Removal of Organic Dyes from Aqueous Solutions by Activated Carbons Prepared from Residue of Supercritical Extraction of Marigold”. The objective of the study was to obtain and characterize a series of activated carbon materials by physical activation of the residue of supercritical extraction of marigold. The effects of carbonization (500 and 700 °C) and activation (700 and 800 °C) temperatures, textural parameters and acid-base character of the adsorbent surface on the sorption properties of the activated carbons were established. The adsorption capacities of the activated carbon samples studied raised with increasing initial concentration of the organic dye in the solvent, which suggests that at low-dye concentrations, their adsorption has a random character, while at high concentrations, the active centers on the adsorbent’s surface are all occupied, saturating the adsorbent. The kinetics of adsorption of organic dyes were found to be described by the pseudo-second-order model.

Słomka-Słupik et al. [21] authored a submission, entitled “Multicomponent Low Initial Molar Ratio of SiO_2_/Al_2_O_3_ Geopolymer Mortars: Pilot Research”. The use of different secondary materials in the mass of a binder or building mortar to perform a multicomponent low initial molar ratio of SiO_2_/Al_2_O_3_ geopolymers was considered in this work. Alkali-activated binders produced with photovoltaic glass powder in 5%; kaolin clay in 15%; ground granulated blast furnace slag in 30%; alumina–lime cement in 30%; and, interchangeably, fly ash from coal combustion in 5%, fly ash from biomass combustion in 5% or granulated autoclaved cellular concrete in 5% are used in this pilot study. The influence of clay dehydroxylation, curing conditions, glass presence and type of waste material was investigated. The aim was to compare the mechanical and material properties of mortars made on standard sand to determine the strength of the binders.

Zhang et al. [22] contributed their research, entitled “Preparation and Basic Properties of Praseodymium-Neodymium-Chromium Containing Imitation Gemstone Glass”. Four types of gem-imitating glasses were prepared by the elemental substitution of praseodymium, neodymium and chromium elements present in rare-earth glass and examined by combining refractive index, density, spectral characteristics and color parameters. All imitation gemstone glasses are transparent, with a refractive index higher than 1.5 and a density up to 2.6 g/cm^3^. The comprehensive performance of the prepared imitation gemstone glasses can be found in the corresponding natural gemstones, which has a certain practical value. These imitation gemstone glasses have numerous characteristics that can meet the jewelry market demand for gorgeous gemstones of middle and low grades.
